# Supplementary material for: Cardiac MR segmentation based on sequence propagation by deep learning
Source: PLoS One. 2020 Apr 9;15(4):e0230415. doi: 10.1371/journal.pone.0230415 (PMC7144953; doi:10.1371/journal.pone.0230415)
Supplement: Attachment — Submitted filename: Response to Reviewers.docx [file pone.0230415.s001.docx]

Reviewer 1:

1: The literature review is not thorough (IMPROTANT). There are lots of related work that have not been mentioned in this paper. For example, the following works were all focusing on cardiac segmentation.

Answer: The relevant part has been revised in the paper.

2. Please discuss the difference in detail between your method and the one in reference [12]

Answer: 1. We use the dice function as a loss function. 2. In addition to adding the ori[i-1] and label[i-1] branches, the ori[i+1] branch is added. 3. We used all the slices of each 3D image.

3. In your first contribution, you mentioned segmentation of a heart, which includes RV, LV and Myocardium. However, in experiments, only myocardium is segmented, why? Can you method segment all anatomies? If so please conduct experiments.

Answer: This method also has a good effect on the segmentation of LV and RV (we conducted experiments on the dataset of West China Medical College, Sichuan University). However, our ACDC dataset does not have split labels for LV and RV, so experiments cannot be performed.

4. Only one dataset (ACDC) was used. I would like to see experiments on different datasets, such as Sunnybrook cardiac data and 2011 LV segmentation challenge.

Answer: I did not successfully get these two data sets. However, I conducted an experiment on the heart data set provided by West China Hospital of Sichuan University. The results of this experiment have been reflected in the paper.

Reviewer 2:

The architecture described in this work is in some way similar to others approaches proposed in the literature exactly in the same context as this paper. For example, in [3] the authors propose a way to introduce contextual information into the NN. And in [1] the authors studied the effects of introducing the original information into the encode path of the u-net. Can the authors discuss the differences between those approaches and the one proposed?

Answer: Our approach combines the contextual sequence information of the image with the appropriate loss function and training strategy.

Did the authors consider using the Dice coefficient or Jaccard as the objective function, instead of the cross entropy ? And why?

Answer: I tried using the dice function as a loss function, but the effect is not as good as cross entropy, so I don't use the dice function.

Given that little training data is available, why are the authors not using data augmentation ?

Answer: Considering the fact that the heart data itself is relatively noisy and the contrast between the target area and surrounding tissue is small, no data enhancement method is used.

5 The paper should describe precisely how the dataset were selected for training, validation and testing.
5.a) Were the dataset split by patients or slices ?
5.b) How many images were used for validation (i.e. training the hyperparameters) ?
5.c) Was the validation dataset the same as the one used for testing or training ?

Answer: It has been explained clearly in the article.

6. Remove or translate the comment to english in Fig. 9

Answer：It has been modified

7. First sentence of the 4 paragraph (line 280) is not clear, it should be rewritten.

Answer：It has been modified
