# Supplementary material for: Cardiac MR segmentation based on sequence propagation by deep learning
Source: PLoS One. 2020 Apr 9;15(4):e0230415. doi: 10.1371/journal.pone.0230415 (PMC7144953; doi:10.1371/journal.pone.0230415)
Supplement: Attachment — Submitted filename: Response to Reviewers1.docx [file pone.0230415.s002.docx]

- give the results on all the annotated structures of the ACDC dataset, with standard deviations in addition to mean values. Has been modified
- give results for the experiments on the second dataset, and a thorough description of it. Has been modified
- perform ablation studies and local evaluation to show and discuss the interest of the method compared to a classic U-Net and to the network in (Zheng et al, TMI 2018). If necessary, the results of the other models could be removed, as they are not related to this comparison but instead reinforce the choice of a U-Net like architecture (they all perform worse than U-Net). Has been modified，but I did not delete the results of the other three comparison methods, because these methods are classic segmentation methods.
- give in a table or a figure the results of the proposed method restricted to local sections of the heart (apex, base, middle) in order to discuss the whereabouts of the improvement. If necessary, this could replace the figures from 4 to 6 which support the same message. Besides, these figures could fit in one to save a lot of room. Has been modified
- Refine the writing and avoiding misleading interpretations of hyper-parameters. Has been modified. The article has been checked several times.
